# A qualitative exploration of the discharge process and factors predisposing to readmissions to the intensive care unit

**DOI:** 10.1186/s12913-017-2821-z

**Published:** 2018-01-05

**Authors:** Uchenna R. Ofoma, Yue Dong, Ognjen Gajic, Brian W. Pickering

**Affiliations:** 1 Division of Critical Care Medicine, Geisinger Medical Center, 100 North Academy Avenue, Danville, PA 17822 USA; 2 Department of Anesthesiology, Mayo Clinic, Rochester, MN USA; 3 Division of Pulmonary and Critical Care Medicine, Mayo Clinic, Rochester, MN USA

**Keywords:** Intensive care, Readmission, Discharge, Transitions of care, Patient safety

## Abstract

**Background:**

Quantitative studies have demonstrated several factors predictive of readmissions to intensive care. Clinical decision tools, derived from these factors have failed to reduce readmission rates. The purpose of this study was to qualitatively explore the experiences and perceptions of physicians and nurses to gain more insight into intensive care readmissions.

**Methods:**

Semi-structured interviews of intensive care unit (ICU) and general medicine care providers explored work routines, understanding and perceptions of the discharge process, and readmissions to intensive care. Participants included ten providers from the ICU setting, including nurses (*n* = 5), consultant intensivists (*n* = 2), critical care fellows (*n* = 3) and 9 providers from the general medical setting, nurses (*n* = 4), consulting physicians (n = 2) and senior resident physicians (n = 3). Principles of grounded theory were used to analyze the interview transcripts.

**Results:**

Nine factors within four broad themes were identified: (1) patient factors – severity-of-illness and undefined goals of care; (2) process factors – communication, transitions of care; (3) provider factors – discharge decision-making, provider experience and comfort level; (4) organizational factors – resource constraints, institutional policies.

**Conclusions:**

Severe illness predisposes ICU patients to readmission, especially when goals of care were not adequately addressed. Communication, premature discharge, and other factors, mostly unrelated to the patient were also perceived by physicians and nurses to be associated with readmissions to intensive care. Quality improvement efforts that focus on modifying or improving aspects of non-patient factors may improve outcomes for patients at risk of ICU readmission.

**Electronic supplementary material:**

The online version of this article (10.1186/s12913-017-2821-z) contains supplementary material, which is available to authorized users.

## Background

Readmissions to the intensive care unit (ICU) are associated with increased cost of care and worse patient outcomes [1, 2], with predominant predisposing factors mostly related to the patient [3–8]. However, non-patient factors such as patient inflow volumes [9], ICU occupancy [10], and clinician decision-making practices [11], have also been suggested. Complex and error-prone care transitions between teams at the time of discharge from intensive care may also contribute to unplanned readmissions [12, 13].

Qualitative studies have enhanced our understanding of the nature of intensive care readmissions. Using in-depth interviews of patients and their care providers regarding the care they received on the general wards after ICU discharge, Russell described two themes relating to ICU readmissions – decreased resources on the general wards, and lack of communication between the ICU and ward staff [14]. This study did not include ICU staff and it was not clear when data saturation occurred. Elliot et al. [15], conducted unstructured interviews of 21 nurses across the ICU, hospital wards and in educational and managerial positions and identified five contributory themes: premature discharge from ICU, delayed medical care at the ward level, heavy nursing workloads, lack of adequately qualified staff and highly demanding patients.

The purpose of this study was to further explore perceptions and gain more insight about readmissions to intensive care from a broader section of care providers.

## Methods

### Study setting

This study was conducted at Mayo Clinic, Rochester, MN, an academic tertiary referral center with 213 ICU beds allocated between two hospital campuses - St. Mary’s Hospital and Methodist Hospital. Accessibility to intensive care beds is excellent with a robust system for overflow between ICUs. The study center has no dedicated step-down units.

### Participants

Nursing and physician providers involved in the direct, or longitudinal hospital care of critically-ill patients were recruited from three ICUs and a general medical floor to which ICU patients were most frequently discharged: a 24-bed medical ICU and a 20-bed vascular/thoracic ICU (both at St. Mary’s Hospital), a 21-bed combined medical/surgical/transplant ICU (Methodist Hospital) and a 36-bed general medical ward (St. Mary’s Hospital) staffed by internal medicine consultants.

Participant recruitment was random, with the sample deliberately weighted towards nursing staff who were more directly involved with processes surrounding discharges and readmissions. For every 3 nurses, we interviewed one consultant physician, one resident physician or one fellow physician. Participants (except consultants) had to have at least one-year experience working in their designated environments. The final sample included nine nurses and ten physicians, consisting of ten providers from the ICU setting (5 nurses, 2 consultant intensivists, 3 critical care fellows), and 9 providers from the general medical setting (4 nurses, 2 consulting physicians and 3 senior resident physicians).

Study participants were read an oral script (Additional file 1) explaining the rationale for the research and the voluntary nature of participation. The study was approved by the Mayo Clinic Institutional Review Board.

### Data collection

All interviews were conducted in a pre-arranged private meeting room in the hospital, using an interview guide (Additional file 2) which sought to explored participants’ work routines and perceptions of the discharge process and ICU readmissions. The interview guide underwent pilot testing with three ICU nurses and three critical care fellows who did not participate in the study. The interviews lasted 30–45 min and were digitally audio-recorded and professionally transcribed. Field notes were also taken during the interview to assist in data analysis and to document concepts for future exploration.

### Data analysis

Data analysis occurred simultaneously with data collection so that emerging concepts could be further explored in subsequent interviews. All interview transcripts were coded using qualitative software for data management (NVivo, QSR International Doncaster, Victoria, Australia). Coding involved reading each transcript and putting like elements of text into broad categories, which were then systematically reviewed to establish core concepts and themes. Data saturation was the primary determinant of how many interviews were conducted. When no new information was gathered for each of the main themes generated, data collection was stopped. After broad themes were identified, all interviews were reviewed again for the presence of each theme and to further characterize the range of responses within each theme. Representative quotes were abstracted during the analytic process and further examined during the manuscript writing process to ensure that these best reflected the interpreted experiences of participants.

## Results

Four main themes were identified. These included patient, process, provider, and organizational themes. Nine factors within these four themes that could potentially lead to readmissions were explored (Table 1). Factors that were cited by the greatest proportion of interview participants related to *communication* (84%) and *discharge decision-making* (79%). A higher proportion of nurses than physicians cited factors relating to *transitions of care* (89% vs 60%) and *discharge decision-making* (89% vs. 70%). Conversely, more physicians than nurses cited factors relating to *undefined goals of care* (90% vs. 60%) and *communication* (100% vs. 67%). Additional quotes for each identified factor are outlined in Tables 2, 3, 4 and 5.

**Table 1:** Frequency of reported factors predisposing to ICU readmissions

Factor	Physicians Interviews *n* (%) *n* = 10	Nurse Interviews *n* (%) *n* = 9	Total Interviews *n* (%) *n* = 19
Patient Factors			
Severity of illness	6 (60%)	5 (56%)	11 (58%)
Undefined goals of care	9 (90%)	5 (56%)	14 (77%)
Process Factors			
Communication	10 (100%)	6 (67%)	16 (84%)
Transitions of care	6 (60%)	8 (89%)	14 (77%)
Provider Factors			
Discharge decision-making	7 (70%)	8 (89%)	15 (79%)
Experience and comfort level	6 (60%)	5 (56%)	11 (58%)
Organizational Factors			
Resource constraints	7 (70%)	3 (33%)	8 (42%)
Institutional policies	4 (40%)	5 (56%)	9 (47%)

**Table 2:** “Patient” factors and illustrative quotes

Factor	Quotes
Severity of Illness	“People are so much sicker because we keep them alive through so much (interventions), and then they have so many more comorbidities. You kind of wonder when are we not really doing a good thing anymore?” *Nurse* “Typically patients get readmitted because they decompensate. Sometimes it’s from the same underlying process that led them to ICU in the first place, especially if they have terrible underlying disease like we have in our (transplant) ICU. There’s a lot of things we can’t fix, so they’ll bounce back and forth relatively often.” *Nurse* “The (transplant) patients are just a sick population. They have the (highest) potential of getting an infection at any time. Those are the most common readmissions (that) we see over and over.” *Nurse*
Undefined goals of care	“There’s a range of complexity on the medical service, and you’re always going to have those patients that are clearly near the end of their life, and any acute issue on somebody with that level of comorbidity could be considered ICU level kind of stuff. I don’t think the right answer is that all those people should be in the ICU (and) I’m not sure it’s to their benefit. (Care providers) need to do better with palliation and everything else.” *Consultant Physician* “For (patient’s) code status, we do a good job, but not always with other aspects of goals of care. This is because sometimes there’s a conflict between the acute issues and the big picture of things. I think we need to be better in communication with the primary team and have more care conferences to discuss goals of care so everyone’s on the same page.” *Nurse* “We have had multiple patients who have terrible underlying disease that there isn’t any cure for, and we’re trying to manage their symptoms, but unless you can cure the underlying problem, you can’t make them better, and so palliative care is something we always try to address in those types of situations if it’s (deemed) futile. (But) if the patient or the family is not ready to (have the discussion), that will frequently lead to multiple readmissions, which we’ve seen on this unit many times.” *Nurse*

**Table 3:** “Process” factors and illustrative quotes

Communication	“A lot of times when we discharge patients, it seems like the nursing-to-nursing report is different than the physician-to-physician report. Nurses are more aware of (things) like when the patient gets up to the commode, they’re very transiently short of breath, whereas physicians might not. So, when the floor nurses now inform the physician about a (transient) desaturation of 80(%), and they’re like, “Well, holy cow, I didn’t get a report on that.” *Nurse* When somebody’s coming down (from the ICU), I’ll occasionally get a call — maybe a third of the time — from the ICU consultant (physician). That’s worthwhile and beneficial for the higher-level stuff (but) it would be more worthwhile (to focus on) the communication between the nurse who’s dismissing the patient (from the ICU) and the nurse who’s accepting the patient (on the floor) because that’s the generator for most of the (ICU readmissions). (For example) “if they get transiently tachypneic, just suction them and they’ll be fine”. That kind of (communication) would be a higher yield. *Consultant Physician* My personal observation is a lot of physician assistants and nurses on the floor are afraid to let the consultant know (of a change in patient status), especially when they don’t want to bother him. (They believe) the consultant thinks it’s a sign of weakness. So, the consultants, most of the time don’t get (real time) information (on) what’s happening. Sometimes the nurse is afraid to communicate with the residents. So, they default to the (emergency response) system (as) a safety net.” *Resident Physician*
Transitions of care	“Sometimes it is difficult to get a bed on some floors and (it complicates) trying to find a time when the receiving nurse and myself can meet up and give report and transfer the patient in a safe manner. If myself and the receiving nurse don’t get a very good hand-off and something gets missed or something of that nature, I guess that could somehow lead to them coming back.” *Nurse*

**Table 4:** “Care provider” factors and illustrative quotes

Discharge decision-making	“Being more cautious in sending our (ICU) patients out (will affect readmissions). We do send patients out quickly. We look at (the patients) and say we’re not doing anything ICU-wise for them and then we’re done. Whereas we may not be giving them a lot of interventions that are ICU-related, they still may warrant some monitoring for longer.” *Nurse* “I think that in general it’s difficult to get patients out of the ICU. (During) rounds, as long as everybody seems to agree from a physician standpoint, it takes a lot for the nurse to be able to convince (the physician) to keep the patient (in the ICU), depending on who is on staff and who (else is on) the team.” *Nurse*
Provider experience and comfort level	“Sometimes the nurses on the floor become uncomfortable with the patients who are per se ‘busy’, whether it’s adjusting to changes or agitation, so they call the emergency response team on these patients and (request) a higher level of care. Sometimes the (emergency response) calls are so repetitive that I think (the patients) just get accepted (into the ICU) because we always go down and assess them.” *Nurse* “(The readmissions) are overwhelmingly usually respiratory related, and the most prominent (cause) anecdotally would be nursing’s discomfort with respiratory issues, triggering the emergency response team as soon as they come to the floor, and they end up right back in the ICU.” *Consultant Physician* “Sometimes we have patients that, any time you get them up to the chair or something, their heart rate goes up to the 120 s or 130 s. That’s how they are. In the ICU, we feel comfortable with it because we see it all the time, and we can monitor very closely. On the floor, however, if a floor nurse sees that, they would be calling the emergency response team who then sends the patient back up to us.” *Nurse*

**Table 5:** “Organizational” factors and illustrative quotes

Resource Constraints	“In this hospital, when you try to find factors that (related to) bounce back to intensive care unit, your results would be largely influenced by the fact that we don’t have an intermediate care unit. So, if you have a patient with chronic atrial fibrillation, and he’s an outpatient with heart rates of 110, 115 — and in this hospital, having a heart rate of 115 without any other symptoms is a criterion to transfer you to the intensive care unit, and we know that there are outpatient physicians who are comfortable managing (atrial fibrillation) in this setting, even with some observation.” *Resident Physician* “Nursing resources (play a role). I don’t know (floor nurses) feel they have the resources to check on those patients who are requiring a lot of respiratory support. They just don’t have the resources, the staffing to check on them as frequently as they feel that they would want to. And perhaps that’s their comfort level as well.” *Consultant Physician*
Institutional Policies	“We had one patient who had an Ivor Lewis (operation) who went out to the floor. He had some delirium in the ICU and just wasn’t quite over it yet. (While) on the floor, (he) pulled out his NG tube, and needed to have the Cortrak® type of NG tube, and so he came back to the ICU just for an NG tube placement.” *Nurse* “A lot of times we’ll get requests to admit a patient for a procedure, like a central line or a paracentesis or a thoracentesis.” *Resident Physician*

### Theme 1: Patient factors

#### Severity of illness

With acute illness often superimposed on chronic comorbidities, ICU patients constitute the sickest group of hospitalized patients and their clinical care is particularly very challenging. The therapeutic effect of the index ICU stay was often not enough to prevent protraction of illness or readmission to intensive care. A fellow physician and a nurse explained: *“The patients are legitimately sick and they either have little chance of full recovery, or their recovery is going to be protracted no matter what treatment(s) we provide. They’re always sort of on the brink of instability, and (they) bounce back to the ICU.” “A lot of (readmissions) has to do with the acuity and the comorbidity factor. (The patients) are very sick to start with. That’s not changeable. You can’t fix that. You can only medically optimize them.”*

#### Undefined goals of care

With critically-ill patients often requiring life-sustaining treatments, a clear articulation of realistic goals of care for individual patients helps to define the need for intensive care and ongoing life support. Study participants indicated that often, this was not the case. They noted instances of patients moving back and forth between the ICU and general ward before goals of care were eventually articulated. A nurse narrated her experience:

*“Prior to ICU admission,* g*oals of care are (often) not discussed. I don’t know how many times we’ve brought someone (to the ICU), only to withdraw (care) the next day. (Goals of care) need to be addressed before we go dropping in central lines and (engaging rapid response teams). You can’t make everything better.”*

Participants acknowledged perceived barriers in having goals of care discussion in the intensive care setting. The time of primary admission to intensive care was probably not the optimal time for such discussions. A resident physician noted: *“There are many times a patient is admitted to intensive care, where the ICU physician is the first to ever bring up the topic of ‘no escalation of care’. It’s very hard to do that in an acute setting with the patient unstable. It’s much better (and) easier to make that kind of decisions when they’re not as unstable.”*

In other instances, family dynamics weighed against such discussions. One resident physician noted: *“It’s (always) a hard conversation. In (one) situation, we had the palliative care (team) involved (but) the (patient’s) family couldn’t come to a decision and went back and forth. In those situations, sometimes it does require an ICU admission to address goals of care, because (physicians) can’t make that decision for them.”*

### Theme 2: Process factors

#### Communication

Effective communication is essential to reduce medical errors in environments like the intensive care unit [16]. Instances of suboptimal communication between members of multidisciplinary care teams were cited by study participants as major contributing factors to intensive care readmissions.

Oftentimes, the environment and atmosphere for efficient hand-off communication between teams at the time of ICU discharge was suboptimal. A fellow physician noted: *“There were situations where the sign-out probably suffered because of the time constraint of being pushed to get the patient out faster. Sign-out was more brief over the phone (and) was done in the middle of rounds. With that, you do have the potential for those transfer summaries and the whole sign-out process to suffer some.”*

Key information that may forestall ICU readmission are not always clearly communicated at the time of ICU discharge. A nurse explained: *“It’s not (always) as good a communication. For example, a patient’s (systolic) blood pressure (may) normally be low. Simply giving that nurse-to-nurse communication when we transfer patients to let them know that although the patient meets emergency response criteria, this is the patient’s normal blood pressure.”*

Sometimes, the substance of communicated content is watered down and compromised by serial hand-offs. A resident physician noted: *“You're in a hard place trying to communicate everything, because you may communicate to one nurse, and there is a change of shift — So you may talk to three or four nurses down the road. There is a ‘broken telephone’. The same (goes with) the resident (physicians). You may communicate to one resident who signs out to another resident.”*

#### Transitions of care

Inefficiencies in care transition between intensive care and general medical settings have been linked to unplanned readmissions. Examples include discharge at times of shift change/nights/weekends [17], and delays in transfer [12, 13]. Study participants alluded to these facts. Regarding discharge at times of shift change, one nurse noted: *“I feel like when I come on shift change at 7:00 p.m. and I have to give a report on a patient that I’ve met for 10 min, it is not good. I don’t know what I should be telling the nurse (on the floor). Should I tell her it’s okay if he desaturates to 80(%) for a few seconds or a few minutes? I don’t know the patient well.”*

Variable wait times to discharge from intensive care after recognition of the need for ICU discharge were also noted. A nurse explained: *“There are times where you’re transferring a patient to a unit that doesn’t have beds available. So, our patients end up sitting in the ICU for longer and longer periods of time. Sometimes it will take all day to get a bed and the critical care service are normally no longer caring for them.”*

### Theme 3: Provider factors

#### Discharge decision-making

Criteria for ICU discharge relate primarily to need for life-supporting treatment or ICU monitoring. Study participants felt that many readmissions were related to premature discharge from intensive care. Premature discharge was thought to be related to subjectivity in the discharge decision-making process and at other times to occupancy pressures in the ICU. A fellow physician and a nurse explained: “*(The decision to discharge) is really a judgment call on the part of the team. Usually the process involves the team’s assessment with the ICU consultant who agrees or disagrees that the patient was ready to go to the floor.” “When our (intensive care) unit is getting full, there is a certain pressure (to discharge patients). And there’s this attitude that they now don’t need this high (level) of acuity, because their vital signs all look great (to the ICU team). (The floor) are very limited and their nursing perception of what is serious (or) what is concerning, is very different.”*

Nursing responders particularly perceived physicians not consciously being in tune with nursing needs of the patient after ICU discharge. A nurse explained: *“On the floor, patients are forced to be independent. When they have to go to the bathroom, they get up to the commode. We don’t let anybody out of bed in the ICU, so you can’t really assess a respiratory status until someone decides to take five steps to the bathroom. (When the patient) gets down to (the floor) and then they have to walk down the hall and get in the shower, it’s a huge difference and these things happen.”*

Physicians and nurses differed in their perceptions of the extent to which nursing opinion factored into the decision-making process. A resident physician explained: *“It’s the team (that makes the discharge decision). We would ask the nurses. Often the nurse input would be kind of the final because they’re with the patients all the time, and are better placed to anticipate what the floor nurses were able to take care of.”*

A nurse however noted: *“I’ve seen instances where (nurses were involved) but it’s not always a team decision. In some instances, the team will ask the nurse’s opinion. I think that’s probably the best model, but oftentimes I’ve seen it be a little bit different where the staff will just decide that he’s ready.”*

#### Provider experience and comfort level

Care providers have different levels of comfort in caring for patients with specific co-morbidities and complexities. Participants felt that provider comfort level may affect the threshold to activate a medical emergency response team leading to intensive care readmission that may otherwise not have occurred. A nurse explained: *“Before discharge, we, as an ICU team, have to feel like we’ve tried to correct everything that we can from an ICU perspective. However, if the nursing staff on the floor don’t feel comfortable, they’ll call the emergency response team frequently to get the patients readmitted, even if there’s nothing that we can do differently other than put them on a cardiac monitor.”*

Sometimes, nurses are not entirely comfortable with primary providers managing specific system problems as this consultant physician noted: *“As far as the patients who have come down who have returned to the unit, I believe those other issues have been respiratory related as well, where they’ve needed to be back on the non-invasive positive pressure ventilation, and the floor nurses were uncomfortable with having us adjust settings. And those are probably the ones that are going back to the unit sooner rather than (later).”*

### Theme 4: Organizational factors

#### Resource constraints

The lack of high-dependency or step-down units implies that there is no buffer zone where intermediate-risk patients can be cared for, making the intensive care the default fallback option. A fellow physician noted: *“(There are) those patients that you're sending from the ICU back to the floor, who you know are probably stable enough to not need intensive care, but they're borderline in terms of their vital signs and thus are high-risk for a bounce back to the ICU. Those would be the perfect patients for a step-down unit. As a surrogate for that, we often send those patients to monitored settings on cardiology, when they may not be cardiac patients or (to) the chest service, and the primary reason for that is that the nursing staff on chest service are more comfortable taking care of patients with complex respiratory needs who don’t necessarily need the ICU.”*

With step-down unit constraints, patients may be better served in the ICU. A nurse explained: *“In our facility, we can either have patients safely taken care of on the general care floor or safely taken care of in the ICU. Could there be something in between? Certainly, but for the way our system is right now, I think (being in ICU) is safest for the patients. So, I would agree that we readmit patients that may be less critical than some of us here in critical care like to take care of but are definitely inappropriate for the floor.”*

The nursing workload on the floors can potentially amplify the effects of lack of a step-down unit, as this nurse described: *“We need a (progressive care unit), Part of the problem is that (patients move from) ICU to general care, you can’t keep some patients in the ICU because they don’t need it, but when we send them to a floor where the workload is heavy, they can’t be monitored like they probably need to be. There’s no happy medium. It’s one extreme to the other.”*

#### Institutional policies

Emergency response teams help to risk stratify patients for ICU admission. Often, their role as gatekeepers may be influenced by institutional expectations. A consultant physician noted: *“We’re (a) closed (ICU) in the sense that there is a specific ICU team that follows a patient and that the floor teams who sent them (to the ICU) don’t really follow them anymore. (On the other hand) I think we’re wide open when the general expectation institutionally is that if anyone on the floor thinks the patient needs to go to the ICU, then the ICU is obligated to accept the patient even if they don’t think it’s appropriate.”*

Often, the readmissions are for procedures, medications or other therapies that are strictly designated by institutional policy for the ICU only; A nurses explained: *“With the new procedural guideline, the patient has to be on a monitor when you give any intravenous medications. You can give for example, 5 milligrams of metoprolol and the patient can go back to the floor within an hour because they only have to be monitored for a short time.”*

## Discussion

This study examined the experiences and perceptions of physicians and nurses regarding ICU discharge and readmissions. We identified nine factors within four broad themes that participants perceived to contribute to readmission of patients after discharge from intensive care. Several of these factors have previously been described in qualitative studies of ICU readmissions [14, 15]. However our study identified additional conceptual themes and factors (e.g. severity of illness, undefined goals of care, discharge decision-making and institutional culture) while providing further insights into previously described themes.

The perceptions of physicians were mostly concordant with those of nursing staff, with the exception discharge decision-making, where physicians and nurses disagreed regarding the extent to which nursing opinion factored into decision-making by ICU physicians. Also, while physicians underscored the subjective nature of the discharge-making process as a cause of premature discharge from the ICU, nurses placed more emphasis on ICU occupancy pressures. With regards to the role of undefined goals of care, physicians were more attuned than nurses, to potential barriers that were usually encountered in adequately articulating these goals.

Participants in this study perceived that severity of illness and undefined goals of care were the predominant patient factors that predisposed to intensive care readmission. It follows logically that sicker patients are more likely to be readmitted. The severity of illness both at the time of ICU admission and discharge is an independent risk factor for readmission [3, 5, 6].

The co-morbidities of a critically ill patient may not be easily modifiable. However, timely discussion of goals of care may represent an opportunity for improvement, especially for those patients with suboptimal  prognoses. Decisions to limit life support are among the most important and difficult clinical decisions encountered by patients, families, and providers, with a host of factors contributing to physician variability in decisions to limit life support [18]. Participants perceived that failure to promptly and adequately articulate appropriate goals of care from the time of hospital admission to the time of index ICU discharge may inevitably lead to unnecessary and perhaps futile readmission to the ICU for certain categories of patients.

Effective communication improves clinical decision-making and is essential for mitigating errors and achieving high-quality clinical outcomes. Poor communication among critical care teams is a contributing factor to adverse events including readmissions to intensive care [16, 19]. Participants felt that a suboptimal atmosphere for efficient team communication often resulted in inadequacies and discrepancies in communicated content at times of team hand-off.

Premature discharge from index ICU admission and other inefficiencies in transitioning patient care from hospital ICUs to general medical settings have been linked to unplanned ICU readmissions [17, 20]. Determining who is ready for ICU discharge is a daily challenge, often based on the subjective intuition of the clinician [21–23]. Traditionally, discharge decisions are made by attending physicians, in collaboration with other members of the ICU care team. Nurses’ reports of nurse-physician collaboration in decision-making at the time of ICU discharge is positively associated with patient outcomes including ICU readmission and hospital mortality [24, 25]. Significant differences in perceptions of nurse-physician collaborative interaction between nurses and physicians as was reported in our study have also been previously reported [26].

Adverse events from latent failures often arise from organizational factors that determine working conditions and institutional policies. For example, the study institution lacked a dedicated step-down units and participants cited this as a possible reason for readmissions that may not have been necessary. A previous Mayo Clinic study of ICU stays suggested that pressures to shorten the ICU length of stay  and lack of a non-ICU for low-risk patients who require monitoring may be causing high ICU readmission rates [27].

### Implications

Organizations may focus their efforts on modifying or improving aspects of non-patient factors that will improve outcomes for patients at risk of ICU readmission, for example by standardizing care processes and improving communication between multidisciplinary care teams. For instance, transitional care programs after discharge from intensive care have been shown to reduce the risk of ICU readmissions [28, 29]. Also, formalizing multidisciplinary input may improve ICU discharge decision-making, while additional training can improve the comfort level of care providers. Engaging institutional stakeholders can also raise awareness to the possible role of organizational factors.

Several risk stratification scores have been proposed as predictors of ICU readmission [30–33]. Predominantly composed of patient-centered factors, these scoring systems have, however, shown limited predictive abilities. The impact of non-patient system factors may explain the poor to modest discrimination and the inability of these models to reduce readmissions during real world implementation [23]. Future research examining intensive care readmissions approached from a systems perspective should evaluate the role of non-patient factors and their relationship to patient factors.

### Limitations

Our study findings reflect the perceptions of care providers in a single tertiary care academic medical center in the United States and are therefore limited in its generalizability. Our study must also be interpreted in the context of a possible discrepancy between provider perceptions and real-life occurrences. It is possible that participants misinterpreted their own experiences and the experiences and intentions of their colleagues. Finally, our sample of interviewed providers was intentionally weighted towards nursing providers. This may have biased our findings towards a nursing viewpoint. Lastly, the interpretation of expressed experiences of study participants may have been biased from the perspective of the physician authors.

## Conclusions

In this single-center qualitative study, several, predominantly, non-patient factors were perceived by physicians and nurses to be related to readmissions to intensive care. These factors are potential targets for quality improvement efforts that are focused on reducing ICU readmission rates.

## Additional files

Additional file 1: Oral script. (DOC 24 kb)

Additional file 2: Interview Guide. (DOCX 12 kb)

## Abbreviations

ICU: Intensive Care Unit

## References

[1] Rosenberg AL, Watts C (2000). Patients readmitted to ICUs* : a systematic review of risk factors and outcomes. Chest.

[2] Kramer AA, Higgins TL, Zimmerman JE (2012). Intensive care unit readmissions in U.S. hospitals: patient characteristics, risk factors, and outcomes. Crit Care Med.

[3] Frost SA, Alexandrou E, Bogdanovski T, Salamonson Y, Davidson PM, Parr MJ, Hillman KM (2009). Severity of illness and risk of readmission to intensive care: a meta-analysis. Resuscitation.

[4] Metnitz PGH, Fieux F, Jordan B, Lang T, Moreno R, Le Gall J-R (2003). Critically ill patients readmitted to intensive care units--lessons to learn?. Intensive Care Med.

[5] Rosenberg AL, Hofer TP, Hayward RA, Strachan C, Watts CM (2001). Who bounces back? Physiologic and other predictors of intensive care unit readmission. Crit Care Med.

[6] Ho KM, Dobb GJ, Lee KY, Finn J, Knuiman M, Webb SAR (2009). The effect of comorbidities on risk of intensive care readmission during the same hospitalization: a linked data cohort study. J Crit Care.

[7] Kaben A, Correa F, Reinhart K, Settmacher U, Gummert J, Kalff R, Sakr Y (2008). Readmission to a surgical intensive care unit: incidence, outcome and risk factors. Crit Care.

[8] Litmathe J, Kurt M, Feindt P, Gams E, Boeken U (2009). Predictors and outcome of ICU readmission after cardiac surgery. Thorac Cardiovasc Surg.

[9] Baker DR, Pronovost PJ, Morlock LL, Geocadin RG, Holzmueller CG (2009). Patient flow variability and unplanned readmissions to an intensive care unit. Crit Care Med.

[10] Chrusch CA, Olafson KP, McMillan PM, Roberts DE, Gray PR (2009). High occupancy increases the risk of early death or readmission after transfer from intensive care. Crit Care Med.

[11] Yoon KB, Koh SO, Han DW, Kang OC (2004). Discharge decision-making by intensivists on readmission to the intensive care unit. Yonsei Med J.

[12] Garland A, Connors AF (2013). Optimal timing of transfer out of the intensive care unit. Am J Crit Care.

[13] Johnson DW, Schmidt UH, Bittner EA, Christensen B, Levi R, Pino RM (2013). Delay of transfer from the intensive care unit: a prospective observational study of incidence, causes, and financial impact. Crit Care.

[14] Russell S (1999). Reducing readmissions to the intensive care unit. Heart and Lung: Journal of Acute and Critical Care.

[15] Elliott M, Crookes P, Worrall-Carter L, Page K (2011). Readmission to intensive care: a qualitative analysis of nurses' perceptions and experiences. Heart Lung.

[16] Reader TW, Flin R, Cuthbertson BH (2007). Communication skills and error in the intensive care unit. Curr Opin Crit Care.

[17] Hanane T, Keegan MT, Seferian EG, Gajic O, Afessa B (2008). The association between nighttime transfer from the intensive care unit and patient outcome. Crit Care Med.

[18] Wilson ME, Rhudy LM, Ballinger BA, Tescher AN, Pickering BW, Gajic O (2013). Factors that contribute to physician variability in decisions to limit life support in the ICU: a qualitative study. Intensive Care Med.

[19] van Sluisveld N, Hesselink G, van der Hoeven JG, Westert G, Wollersheim H, Zegers M (2015). Improving clinical handover between intensive care unit and general ward professionals at intensive care unit discharge. Intensive Care Med.

[20] Snow N, Bergin KT, Horrigan TP (1985). Readmission of patients to the surgical intensive care unit: patient profiles and possibilities for prevention. Crit Care Med.

[21] Heidegger CP, Treggiari MM, Romand JA, Swiss ICUN (2005). A nationwide survey of intensive care unit discharge practices. Intensive Care Med.

[22] Shear LM, Steingrub JS, Teres D, Shear L (1988). Icu patient triage ranking - a flawed practice. Crit Care Med.

[23] Ofoma UR, Chandra S, Kashyap R, Herasevich V, Ahmed A, Gajic O, Pickering BW, Farmer CJ (2014). Findings from the implementation of a validated readmission predictive tool in the discharge workflow of a medical intensive care unit. Ann Am Thorac Soc.

[24] Baggs JG, Ryan SA, Phelps CE, Richeson JF, Johnson JE (1992). The association between interdisciplinary collaboration and patient outcomes in a medical intensive care unit. Heart Lung.

[25] Baggs JG, Schmitt MH, Mushlin AI, Mitchell PH, Eldredge DH, Oakes D, Hutson AD (1999). Association between nurse-physician collaboration and patient outcomes in three intensive care units. Crit Care Med.

[26] Miller PA (2001). Nurse-physician collaboration in an intensive care unit. Am J Crit Care.

[27] Afessa B, Keegan MT, Hubmayr RD, Naessens JM, Gajic O, Long KH, Peters SG (2005). Evaluating the performance of an institution using an intensive care unit benchmark. Mayo Clin Proc.

[28] Niven DJ, Bastos JF, Stelfox HT (2014). Critical care transition programs and the risk of readmission or death after discharge from an ICU: a systematic review and meta-analysis. Crit Care Med.

[29] Stelfox HT, Bastos J, Niven DJ, Bagshaw SM, Turin TC, Gao S (2016). Critical care transition programs and the risk of readmission or death after discharge from ICU. Intensive Care Med.

[30] Frost SA, Tam V, Alexandrou E, Hunt L, Salamonson Y, Davidson PM, Parr MJA, Hillman KM (2010). Readmission to intensive care: development of a nomogram for individualising risk. Crit Care Resusc.

[31] Gajic O, Malinchoc M, Comfere TB, Harris MR, Achouiti A, Yilmaz M, Schultz MJ, Hubmayr RD, Afessa B, Farmer JC (2008). The stability and workload index for transfer score predicts unplanned intensive care unit patient readmission: initial development and validation. Crit Care Med.

[32] Ouanes I, Schwebel C, Francais A, Bruel C, Philippart F, Vesin A, Soufir L, Adrie C, Garrouste-Orgeas M, Timsit JF (2012). A model to predict short-term death or readmission after intensive care unit discharge. J Crit Care.

[33] Badawi O, Breslow MJ (2012). Readmissions and death after ICU discharge: development and validation of two predictive models. PLoS One.

