# Supplementary material for: A qualitative exploration of the discharge process and factors predisposing to readmissions to the intensive care unit
Source: BMC Health Serv Res. 2018 Jan 5;18:6. doi: 10.1186/s12913-017-2821-z (PMC5755430; doi:10.1186/s12913-017-2821-z)
Supplement: Supplementary file 1 — Oral script. (DOC 24 kb) [file 12913_2017_2821_MOESM1_ESM.doc]

Oral script

My name is Uchenna Ofoma, a Clinical Fellow with the Division of Pulmonary and Critical Care Medicine.

I have approached you because my co-investigators and I are conducting a research project on the phenomenon of ICU readmissions and as a provider in one of the Mayo ICUs, we are hoping to get your insights on this issue.

If you agree to participate, I will be asking your opinion concerning ICU readmissions, you do not have to answer a question if you feel uncomfortable with it.

Your selection is completely random and was not based on any pre-specified criteria. Your participation is completely voluntary. Your current and future employment, education and/ or medical care at Mayo Clinic will not be affected by whether or not you participate. Specifically, your care will not be jeopardized if you choose not to participate. For participating in this interview, you will receive $75 through direct payroll deposit.

The interview will be recorded and transcribed later on for analysis. You will not be required to state your name or any identifiable information. Your responses will also not be tied to any identifiable information. This interview will last approximately 45 minutes

For further details about this study, you can contact the PI Dr Brian Pickering

At phone number 507-255-6276
