# Supplementary material for: A qualitative exploration of the discharge process and factors predisposing to readmissions to the intensive care unit
Source: BMC Health Serv Res. 2018 Jan 5;18:6. doi: 10.1186/s12913-017-2821-z (PMC5755430; doi:10.1186/s12913-017-2821-z)
Supplement: Supplementary file 2 — Interview Guide. (DOCX 12 kb) [file 12913_2017_2821_MOESM2_ESM.docx]

**INTERVIEW GUIDE**

1. Tell me about your typical day in the ICU/Floor
   1. How is your day organized?
      1. Is it always the same?
         1. Probe: If not, what dictates the organization?
      2. Please give examples
   2. Who organizes your day?
      1. Is it always the same person/role of a person?
         1. Probe: If not how and why is that the case?
      2. Examples, examples
   3. How big is your team?
      1. Probe: Who is on your team? (not names, just roles and titles)
2. Tell me about a discharge in which you were involved that went smoothly and as planned
   1. Walk me through the whole process from beginning to end (details and examples)
      1. Probe: What was your role in the process?
      2. Probe: How many staff members were involved in the process?
      3. Probe: How long did this discharge process take?
   2. Were there relatives involved in the process
      1. If yes: What role did they play in the process?
      2. Probe: Pros and cons of having relatives involved
3. Describe a discharge process that did not go so well
   1. Walk me through the whole process from beginning to end (details and examples)
      1. Probe: What was your role in the process?
      2. Probe: How many staff members were involved in the process?
      3. Probe: How long did this process take?
   2. Were there relatives involved in the process?
      1. If yes: What role did they play in the process?
      2. Probe: Pros and cons of having relatives involved
4. Why do you think patients get readmitted to the ICU?
   1. Can you give me an example of why you think this is the case?
   2. Have you seen a common pattern in reasons for readmissions?
   3. How do you think we can reduce readmissions to the ICU?

Depending on the answers they give, they may or may not be prodded further to elucidate on specific points using probing questions like:

Please explain further

What exactly do you mean?

Tell me more about that

Why do you think that happened?

Give me an example from your own experience
